# Effect of Tie Bars on Axial Compressive Behavior of Round-Ended Rectangular CFST Stub Columns

**DOI:** 10.3390/ma15031137

**Published:** 2022-02-01

**Authors:** Zhigang Ren, Qi Li, Chuang Liu

**Affiliations:** 1 School of Civil Engineering and Architecture, Wuhan University of Technology, Wuhan 430070, China; renzg@whut.edu.cn (Z.R.); whutliuchuang@163.com (C.L.); 2 Yichang Urban Construction Investment Holding Group Co., Ltd., Yichang 443000, China

**Keywords:** concrete-filled steel tube, round-ended rectangular, tie bar, confined effect, bearing capacity, ductility

## Abstract

Round-ended rectangular concrete-filled steel tube (RRCFST) columns are prone to local buckling that are close to straight steel plates when used as piers of a bridge and affect its long-term use. In order to solve this problem, tie bars were used in this research to stiffen RRCFST columns. Eleven specimens with tie bars and three specimens without tie bars were tested to analyze influences of cross-sectional aspect ratio, longitudinal spacing, limb numbers and diameter of the tie bar on failure model, confined effect, bearing capacity and ductility of RRCFST stub columns. Finite element models (FEM) with different concrete constitutive models for rectangular and circle parts were established and validated to reveal the mechanism of the constrained effect of tie bars. Experimental and FEM results show that the local buckling scope was decreased and gradually moved to middle height with decreased longitudinal spacings tie bars. The addition of tie bars in RRCFST columns with large aspect ratios slightly enhanced the ultimate bearing capacity, the diameter of tie bars changing from 8 mm to 12 mm greatly enhanced displacement and energy ductility by 58.4% and 85.1%, respectively. However, more tie bars (e.g., two or three limbs) utilization could not further improve the bearing capacity and failure mode. While, the tie bars had very limited contribution to bearing capacity and ductility for RRCFST columns with small aspect ratios, because the outer steel tubes already individually provided for enough confinement on inner concrete. By considering different concrete confined models for rectangular and round-ended parts, an analytical model was proposed and validated to predict the ultimate bearing load for RRCFST stub columns with tie bars.

## 1. Introduction

As a relatively new type of structural member, concrete-filled steel tubes (CFSTs) have lots of advantages, such as good bearing capacity, high seismic property, lightweight, and fast construction speed [1]. CFST has multiple cross-sectional forms, e.g., rectangle [2,3], round-ended rectangle [4,5] and circle [6,7]. The confinement effect of steel tubes on core concrete presents the characteristic of continuity with this order [8]. Round-ended rectangular CFST (RRCFST) column could be suggested for architectural [9] and aesthetical purposes [10]. RRCFST columns also own a comparable axial bearing capacity to the circular one [11] and excellent bending strength on the major axis like the rectangular one. Furthermore, RRCFST column has reduced water flow resistance and enhanced impact resistance by the round-ended part in the bridge pier [12]. However, local buckling could still occur at the outside of the RRCFST column due to the ineffective confined of the steel tube and the shear failure of infilled concrete [13], thus significantly reducing the bearing capacity and ductility [14].

**Figure 1:**
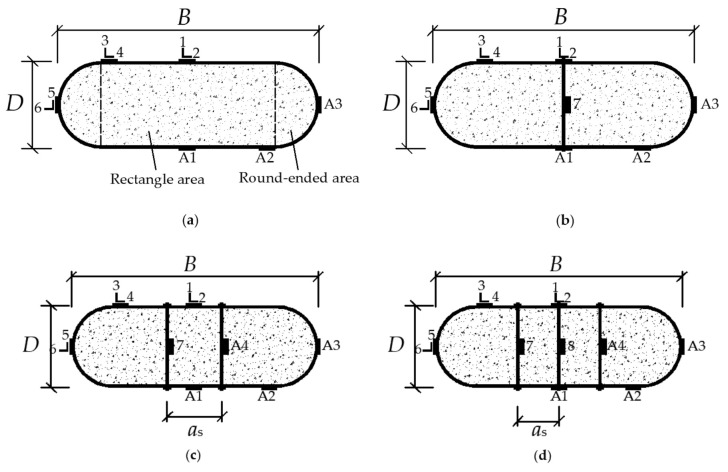
The dimension and the setting of tie bars of the specimens: (**a**) Non-tie bars; (**b**) Single limb of tie bars; (**c**) Two limbs of tie bars; (**d**) Three limbs of tie bars.

Longitudinal stiffeners can help delay the local buckling and increase the bearing capacity of CFST columns [15], but have less influence on the ductility. To adequately use the property of materials under axial load [16] and enhance the ductility [17], a stiffening method of tie bars set on square CFST column was proposed by Cai [18], which effectively delay the local buckling by enhancing the confined effect of the steel tube on concrete, and was more available for rectangular CFST [16]. The lateral deformation of the steel tube was controlled by tie bars [19], and the longitudinal stress distribution of concrete in the column section was transferred more reasonably [20,21]. It is worth pointing out that the RRCFST can be considered as a composite of circular CFST and rectangular CFST. For circular CFST columns, tie bars strengthen the cross-section bonding between concrete and steel tube but slightly increase the axial compression bearing capacity of the column due to the fine confined effect of steel tube [22]. For rectangular CFST columns, the effective restraint area of concrete from the four corners transfer to both sides of the tie bar [23]. The finite element model (FEM) incorporating reasonable constitutive models of the constrained concrete and the steel tube should be developed to figure out the confined mechanism of tie bars for the RRCFST. In general, round-ended rectangular cross-sections are divided for a rectangular part and two round-ended parts (as shown in Figure 1a), which use different constitutive models [13,24] to simulate different confined effects of steel tube on these two parts.

The circular confined concrete constitutive model [25] can be used for simulating mechanical properties of concrete at round-ended part. However, the constraints on the core concrete in the rectangular part are affected by tie bars, which is similar to the mechanical mechanism of rectangular and square CFST columns with tie bars. Liang [26] and Han [25] et al. have proposed constitutive model of concrete for rectangular CFST column based on the experimental results. In addition, a constitutive model of concrete for square CFST with tie bars was proposed by Cai [27], which is based on Mander’s research [28]. As a homogeneous material, ideal elastoplastic model [21,29,30] could be used for simulating steel properties under compression and tension. While, in the situation of large deformation, the strain of steel will go beyond the yield stage and enter the plastic strengthening stage. Thus, a secondary order plastic flow model with a whole stage of stress–strain was proposed by Liang [31] and has been used in many simulations [5,13,24,32,33].

Furthermore, research on analysis model by considering the restraint mechanism of tie bars will help to predict the axial bearing capacity of RRCFST more accurately. For RRCFST columns, a unified calculation method for the concrete stress–strain relationship under uniaxial compression was proposed in reference [33], the applicability and accuracy of the method were proved [13]. The bearing capacity of CFST column was affected by the triaxial effects produced from the confinement of steel tube on core concrete [16]. Therefore, the confined effect should be considered in the ultimate bearing load calculation of the RRCFST columns with tie bars. The restrained strengthen of concrete is different in different cross-sections. For circular cross-sections [22], the confinement strength (*f*_cc_) of concrete is equal in terms of all directions of the cross-section, and can be obtained by equilibrium relationship. For rectangular cross-sections, the failure criterion for concrete under true triaxial compression is used for calculating *f*_cc_, with a complied calculating process, which is presented in Chinese code GB50010-2010 [34]. As different combinations of multiple rectangular cross-sections, *f*_cc_ for L [35] and T-shaped [36] cross-sections are acquired by the same calculation method of rectangular one. Reference [37] proposed a calculation method for axial compression of RRCFST by transferring the round-ended cross-section to polygonal one.

Many researches have studied the performance of RRCFST, but there have been few investigations on stiffening techniques to prevent its buckling. Moreover, the stiffening mechanism of tie bars is still unclear. Thus, this study quantified the effect of tie bars on the axial compression performance of RRCFST stub columns through experimental and numerical analysis. Compressive tests are conducted on 14 specimens with various designed aspect ratios of cross-sections, longitudinal spacing, limb number and diameter of the tie bar. The influences of such parameters on the local buckling, failure model, bearing capacity and ductility are analyzed and discussed. Additionally, reasonable constitutive models of concrete and steel tube are applied for establishing FEM, and the results of the FEM give further insight into the behaviors of the proposed columns. Based on the confined model from references and stress contour from FEM, a calculation model of ultimate bearing load for the RRCFST column with tie bars is proposed.

## 2. Research Methods

### 2.1. Specimens Preparation

Figure 1 presents specimens without tie bars and three ways for setting tie bars. Besides, six strain gauges are setting at the middle section of the column to test the strain of specimens, of which 1, 3, 5 for the axial strain, and 2, 4, 6 for the lateral strain, and 7, 8 for the strain of tie bars. Symmetrical strain gauges A1, A2, A3, A4 are setting for preventing errors due to the initial defect.

**Figure 2:**
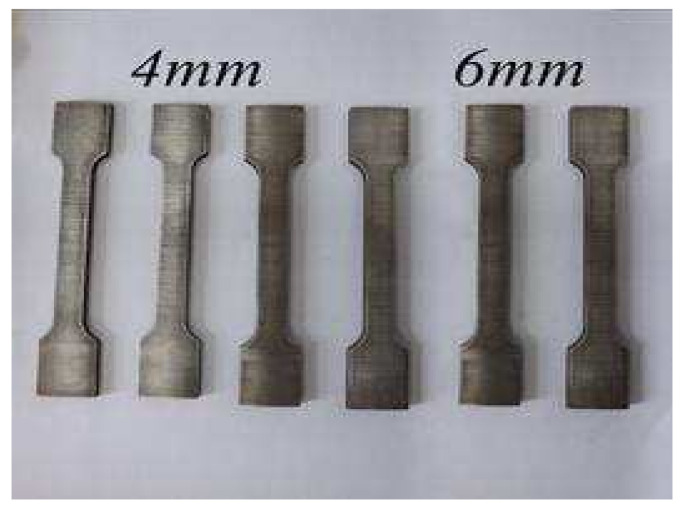
Steel specimens for tensile test.

Fourteen RRCFST columns based on three designed aspect ratios (*B*/*D* = 1.5, 2, 3, of which *B* and *D* are the length and width of the cross-section, respectively, and the steel tubes produce errors in dimension after fabrication, and *D* and *B* in this paper from the actual measured value), height (*L*) of the cross-section, the thickness of the steel tube (*t*), the limb number (*n*), the diameter (*d*), the longitudinal (*b*_s_) and the horizontal (*a*_s_) span of tie bras were experimentally studied by axial compressive test. This main parameter of RRCFST columns and the sectional steel ratio (*α*_s_) are shown in Table 1. According to the Chinese code GB/T228-2002, *Room temperature tensile test method for metallic materials* [38], the specimens in Figure 2 were used for the tensile test and the tested results of basic mechanical properties are shown in Table 2, including yield strength (*f*_y_) and ultimate tensile strength (*f*_u_) of steel tubes, ultimate tensile strength (*f*_bu_) of tie bars, modulus of elasticity (*E*_s_), and passion ratios (*γ*).

The Grade 42.5 Portland cement, granite stones, medium sand, tap water were used as raw materials to prepare the concrete. The average axial compressive strength (*f*_c_) of concrete is 35.21 MPa by testing six standard cubic samples, and the modulus of elasticity (*E*_c_) is 24341.85 MPa.

### 2.2. Experimental Method

**Figure 3:**
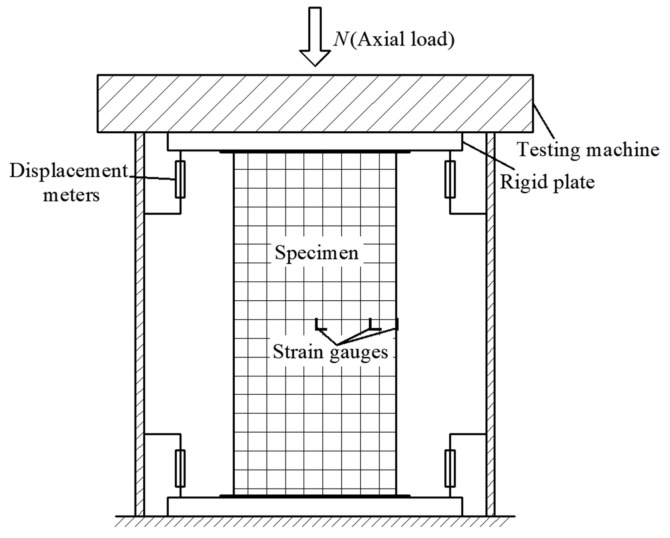
Compressive test setup.

The compressive test setup is presented in Figure 3. The deflection of column was measured by four displacement meters at the supports. The local buckling was observed and the corresponding load was recorded. The axial and lateral strains of steel tubes were measured by strain gauges, as shown in Figure 1. The 15% of the estimated ultimate load was exerted 2–3 times as a preload. Then, the loading regime was controlled under deflection with 0.02 mm/s at initial stage, and the loading rate was reduced to 0.01 mm/s when the force was near the peak load until failure. The testing program was stopped when the three following phenomena occurred: (1) residual bearing capacity is less than 50% of peak load; (2) seam welds were cracked; (3) axial deflection reached 4% height of columns.

### 2.3. Finite Element Analysis

Based on the experimental material properties, a finite element model (FEM) in ABAQUS software was adopted in this research to further study the mechanical performance of RRCFST columns.

#### 2.3.1. Simulation of Concrete

**Figure 4:**
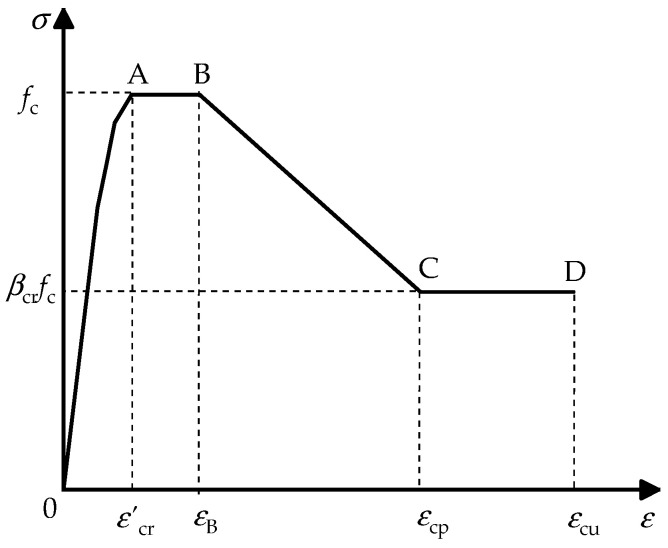
Constitutive model of concrete at rectangular area.

The 3D eight-node solid element C3D8R was selected for concrete. By several trials of numerical analysis, the different constitutive models of concretes at rectangular and round-ended areas were used in the FEM, which could be better validated by the experimental results. The divided areas are shown in Figure 1a. In this study, the constitutive model of concrete at the round-ended area under compression was like that of circular CFST in reference [25]. With the modified coefficient of tie bar (ζ) from Cai [27], the constitutive model of concrete at rectangular area under compression from Liang [26] was adopted and is shown in Figure 4.

At elastic stage, the lateral deformation of concrete is small and the confinement effect of tie bars is inefficient. Hence, the ascent stage (OA in Figure 4) of constitutive model of concrete can be expressed by Mander’s Formulas (1)–(4) [28].

(1) $$\sigma_{c}=\frac{f_{c}\lambda (\frac{\varepsilon_{c}}{\varepsilon_{cr}^{\prime }})}{\lambda -1+(\frac{\varepsilon_{c}}{\varepsilon_{cr}^{\prime }})}$$

Of which:

(2) $$\lambda =\frac{E_{c}}{E_{c}-(\frac{f_{c}^{}}{\varepsilon_{cr}^{\prime }})}$$

(3) $$E_{c}=3320\sqrt{f_{c}}+6900$$

(4) $$\varepsilon \prime_{cr}=\left\{\begin{array}{ll} 0.002 & f_{c}\leq 28\;\mathrm{MPa}\\ 0.002+\frac{f_{c}-28}{54,000} & 28<f_{c}\leq 82\;\mathrm{MPa}\\ 0.003 & {\;f}_{c}>82\;\mathrm{MPa} \end{array}\right.$$

where, *σ*_c_, *ε*_c_ are the axial stress and strain of the concrete, respectively; *ε*’_cr_ is the peak strain of concrete, between 0.002 and 0.003;

Liang’s formulas [26] are expressed by Formula (5) for AB, BC and CD.

(5) $$\sigma_{cr}=\left\{\begin{array}{ll} f_{c} & \varepsilon \prime_{cr}<\varepsilon_{c}\leq \varepsilon_{B}\\ \beta_{cr}f_{c}+\frac{\left(\varepsilon_{cp}-\varepsilon_{c})(f_{c}-\beta_{cr}f_{c}\right)}{\left(\varepsilon_{cp}-\varepsilon_{B}\right)} & \varepsilon_{B}<\varepsilon_{c}\leq \varepsilon_{cp}\\ \beta_{cr}f_{c} & \varepsilon_{c}>\varepsilon_{cp} \end{array}\right.$$

where, *ε*_B_ and *ε*_cp_ are strains corresponding to point B and C in Figure 4, and equal 0.005 and 0.015, respectively; *β*_cr_ is the parameter for *σ*–*ε* curve of concrete at the declined stage. According to reference [27], *β*_cr_ is determined by the following equations:

(6) $$\beta_{cr}=\left\{\begin{array}{ll} 1.0 & \frac{D}{t}\leq 24\\ \sqrt{1+\xi }\;\cdot \sqrt{1+2\zeta }\cdot (2.5-\frac{1}{48}\cdot \frac{B}{t}) & 24<\frac{D}{t}\leq 48\\ 0.5 & \frac{D}{t}>48 \end{array}\right.$$

Of which:

(7) $$\xi =\frac{A_{s}f_{y}}{A_{c}f_{ck}}$$

(8) $$\zeta =\frac{n_{b}A_{b}f_{ly}}{(B-D)b_{s}f_{ck}}$$

where, *ξ* and *ζ* are the constrain coefficients of steel tubes and tie bars, respectively; *A*_b_ is the cross-sectional area of single tie bar; *f*_ck_ is the standard value of axial compressive strength of concrete, and take value according to the Chinese code GB-50010-2010 [34]; *f*_ly_ is the yield stress of tie bar. Hardened steel was used to produce tie bars in this study, and the *f*_ly_ = *σ*_0.2_, namely stress value corresponding to 0.2% plastic strain.

#### 2.3.2. Simulation of Other Materials

The 3D four-node shell element S4R was selected to simulate a steel tube. The secondary order plastic flow model was used as the constitutive relationship of steel tube [25], which could accurately describe the mechanical behavior of steel tube under axial and radial pressures and tension. The stress–strain curve of this model includes elastic stage, elastoplastic stage, plastic stage (yield stage), strain hardening stage and final plastic deformation.

“Surface-to-surface” was used to simulate the interaction between concrete and steel tube. Penalty function with friction coeffective of 0.5 was used for the tangential behavior, and “Hard” contact for the normal behavior.

The 3D two-node linear truss element T3D2 was selected to simulate the tie bar. Ideal elastoplastic model was used to reflect the stress–strain relationship of tie bars. “Embedded region” was selected to simulate the interaction relationship between tie bars and concrete, and “Tie” for tie bars and steel tube.

### 2.4. Finite Element Model and Verification

**Figure 5:**
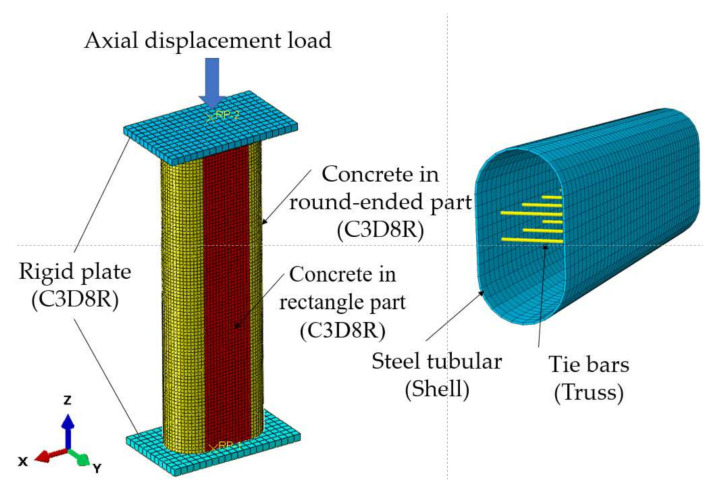
Finite element model of RRCFST.

Figure 5 exhibits the finite element model of the RRCFST column. To get stable outputs, the mesh size was set around 10–20 mm for concrete and 20–25 mm for steel tube by considering both calculation speed and accuracy. According to the experimental loading method, two steel plates with high stiffness were set on both ends of column. An ultimate displacement from experiment was loaded on the reference point, which was coupled to the steel plate at one end of the column, and the degrees of freedom in all directions were restrained at another end of the column.

The experimental (EX) and finite element model results (FE) of bearing capacity (*N*_u_) are compared in Table 3. The average ratio of FE/EX is 1.03, which demonstrates a good agreement between FE and EX results. *SI* is the strength index and could describe the material strength utilization efficiency of specimens, as presented by Equation (9).

(9) $$\mathrm{SI}=\frac{N_{u}}{A_{s}f_{y}+A_{c}f_{c}}$$

**Figure 6:**
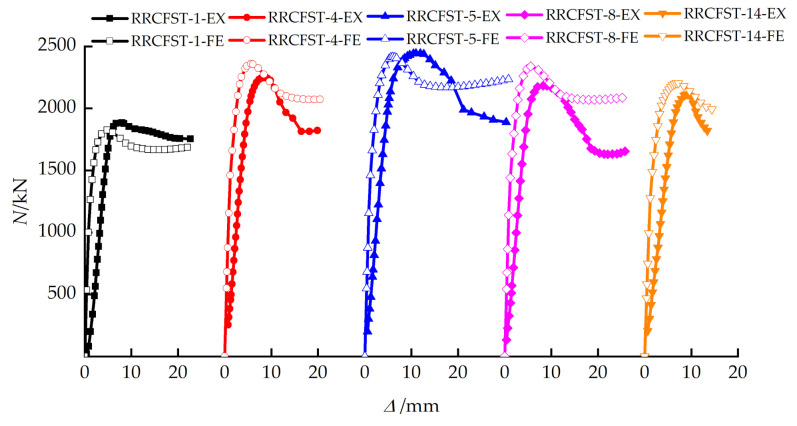
Comparison of load curves for part of the test column by FE analysis and the experiment.

Figure 6 provides comparisons of load-deflection curves of partial specimens, and similar trends of deflection against load can be observed. The stiffness from finite element model was larger due to the homogeneous material assumption without any imperfection. The initial defects of experimental specimens like insufficient seam welds of steel tube and initial micro-cracks of concrete were not taken into consideration in the finite element model.

## 3. Results and Discussions

### 3.1. Failure Modes

**Figure 7:**
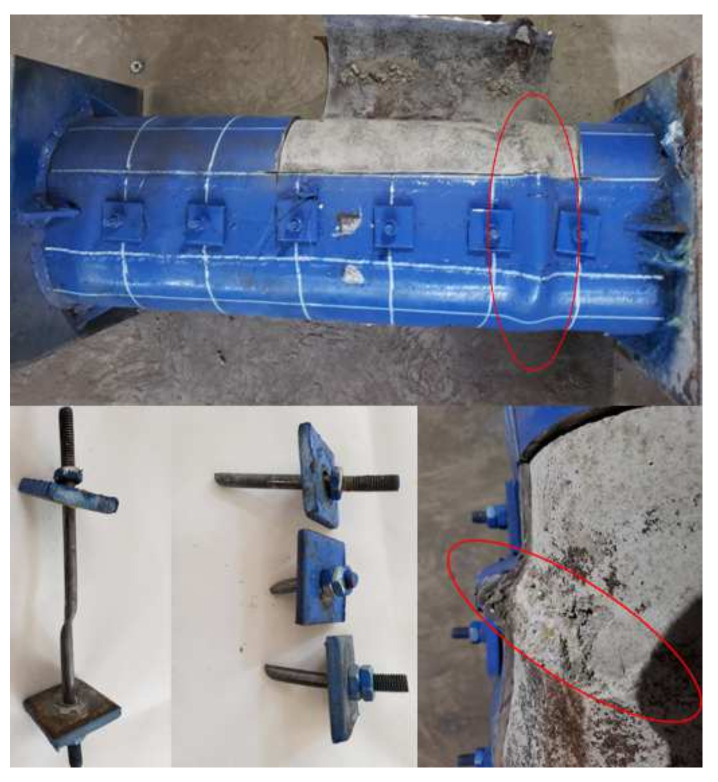
Typical failure modes of concrete and tie bar.

At the initial loading stage, each RRCFST column is in an elastic state with small deformation, and local buckling occurs on the specimens near the ultimate loading condition. Figure 7 shows a typical failure mode of the RRCFST specimen. The inner concrete has a brittle shear failure at the position of local buckling occurs. Tie bars are subjected to partial shear stress, which are broken and fails under the combined action of tension and shear.

**Figure 8:**
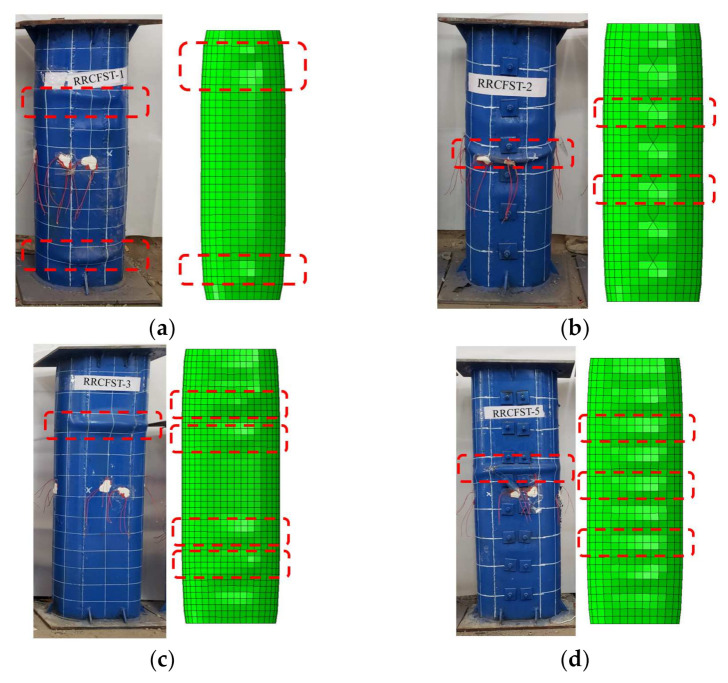

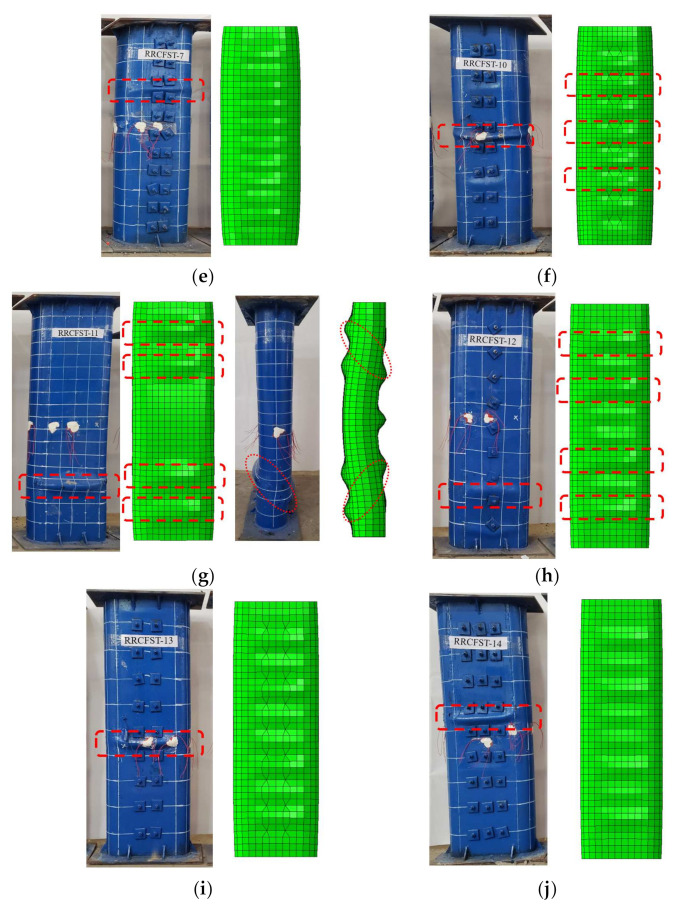
Failure modes of partial specimens: (**a**) RRCFST-1; (**b**) RRCFST-2; (**c**) RRCFST-3; (**d**) RRCFST-5; (**e**) RRCFST-7; (**f**)RRCFST-10; (**g**) RRCFST-11; (**h**) RRCFST-12; (**i**) RRCFST-13; (**j**) RRCFST-14.

Figure 8 shows the failure modes of part of RRCFST specimens in the experiments and FEM. The columns with a designed aspect ratio of 1.5 (Figure 8a,b) are close to circle CFST with better confinement effect; the local buckling occurs after the ultimate load with a lower buckling amplitude compared to other columns.

Figure 8c–f shows the failure modes of columns with designed aspect ratio of 2 and different parameters of tie bars. RRCFST-3 has a larger aspect ratio and results in the larger local buckling range than RRCFST-1. RRCFST-7 has smaller longitudinal spacing for tie bars and presents a narrower range of local buckling than RRCFST-5. By increasing the diameter of tie bars, the RRCFST-10 shows similar failure mode than RRCFST-5. The different failure modes indicate that the local buckling can be partly compensated by reducing the longitudinal spacing of tie bars but has less influence by the diameter of tie bars.

In Figure 8g, RRCFST-11 has the highest designed aspect ratio and leads to the largest confinement difference between the rectangular and round-ended parts. The steel rigidity of the rectangular region is insufficient and results in lateral flexure. The local buckling of RRCFST-11 occurs before the peak load and the strength of the steel tube is inadequately used. With the addition of tie bars, the lateral stiffness of the column is enhanced and the lateral flexure of the specimens disappears, as shown in Figure 8h. With the increase of the tie bars limb numbers as seen in Figure 8i–j, the local buckling replaced from end to middle section, but failure modes changed not obvious. This phenomenon indicates that a tie bar with a single limb can improve the lateral stiffness of the column and change its buckling mode, but continue increasing the limb number of the tie bar could not produce further improvement. This reveals the similar influence of the limb number of tie bars on the bearing capacity, which will be discussed in Section 3.3.

### 3.2. Confined Effect

**Figure 9:**
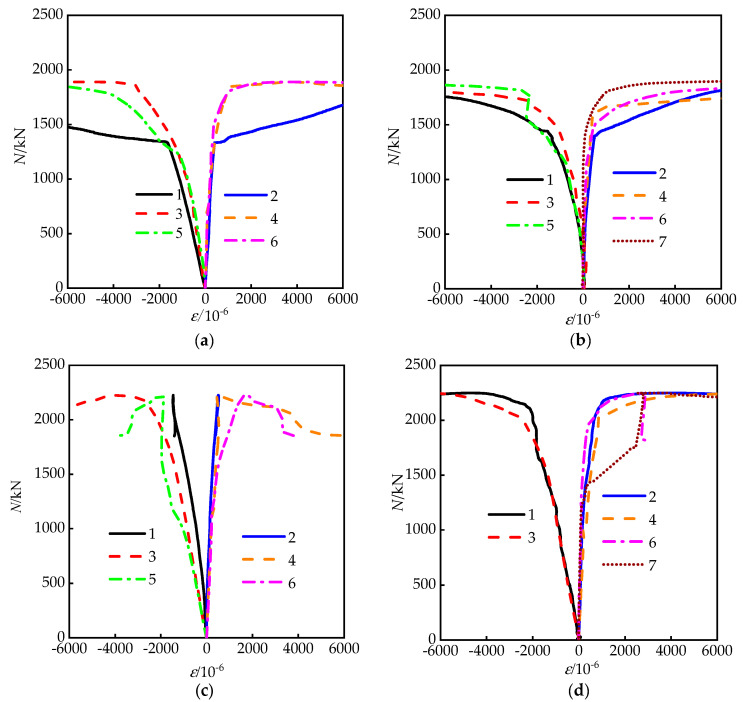

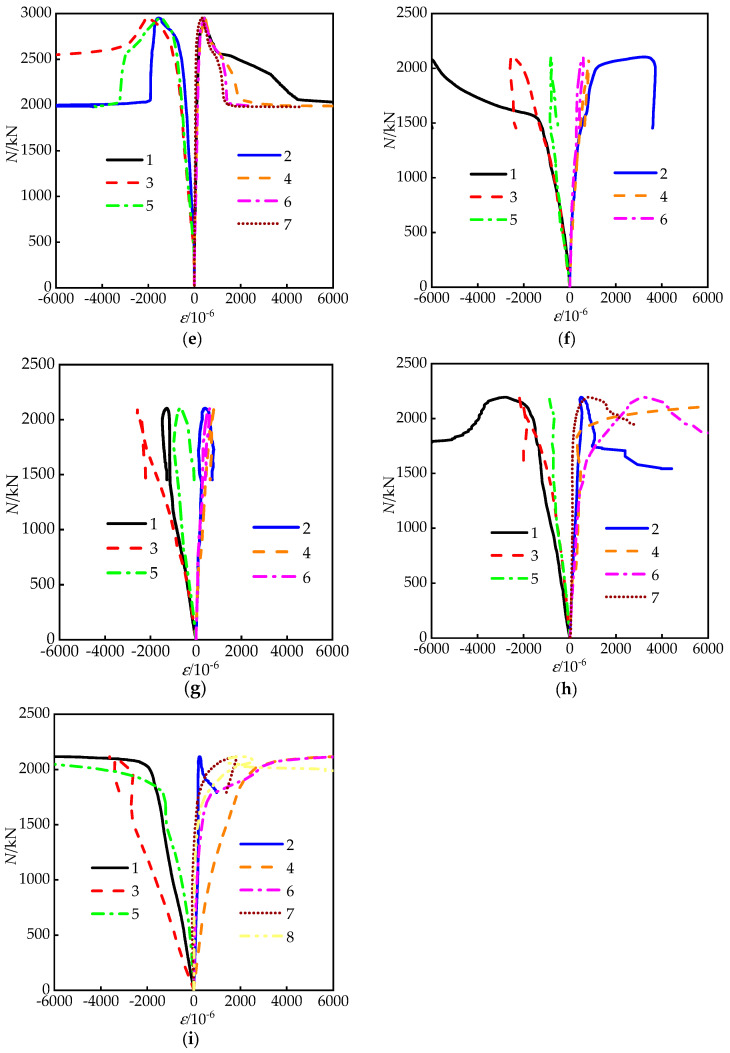
Strain curves of the steel tube at different measure point and direction: (**a**) RRCFST-1; (**b**) RRCFST-2; (**c**) RRCFST-3; (**d**) RRCFST-4; (**e**) RRCFST-5; (**f**) RRCFST-11; (**g**) RRCFST-12; (**h**) RRCFST-13; (**i**) RRCFST-14.

Figure 9 shows load–strain curves of partial specimens; the strain measuring points can be seen in Figure 1. The negative and positive values represent longitudinal and lateral strains of the steel tube, respectively. For RRCFST columns with low designed aspect ratio (e.g., 1.5), the tie bars have no obvious effect on the strain of the steel tube, probably because of the already existing good confinement from the steel tubes. While, for the RRCFST columns with a larger aspect ratio, as shown in Figure 9c,f, the development of longitudinal strains far exceeds the lateral strains. The longitudinal strain reaches the yield strain of the steel tube, while the transverse strain is still at a lower value, and the restraint effect of the steel tube was not fully exerted. As shown in Figure 9d–e,g–i, the development of longitudinal and transverse strains is relatively consistent. The axial strain of tie bars is close to the transverse strain of steel tubes, indicating that the restraining effect of steel tubes on inner concrete is obviously improved by tie bars.

**Figure 10:**
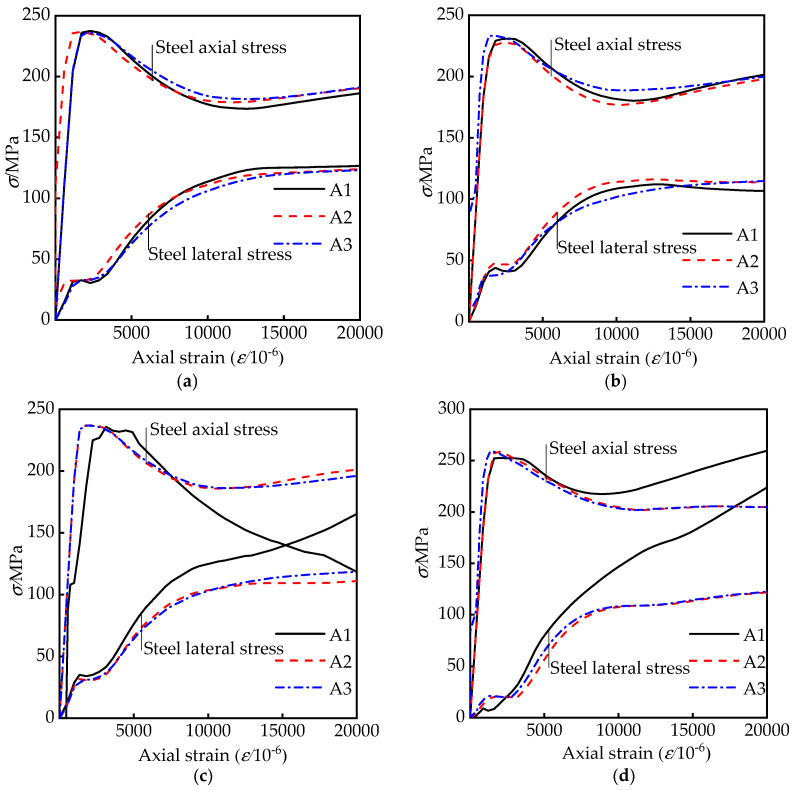
Comparisons of stress–axial strain curves of steel tube: (**a**) RRCFST-1; (**b**) RRCFST-3; (**c**) RRCFST-4; (**d**) RRCFST-13.

To give further insight on the influences of geometrical sizes of column and tie bars on the restraint effect of steel tubes, some stress–strain curves from FEM analysis at each measuring point are demonstrated in Figure 10. Depending on the research in reference [13], the efficient confinement effect is reflected by the separates between the lateral and longitudinal stress–strain curves. Figure 10a,b shows that the separates between the curves of two directions are raised with the increasing aspect ratios, and lead to a reduced confinement effect. The confined effect of the steel tube in the rectangular area is obviously increased after adding the tie bars, even two curves are crossed in Figure 10c.

**Figure 11:**
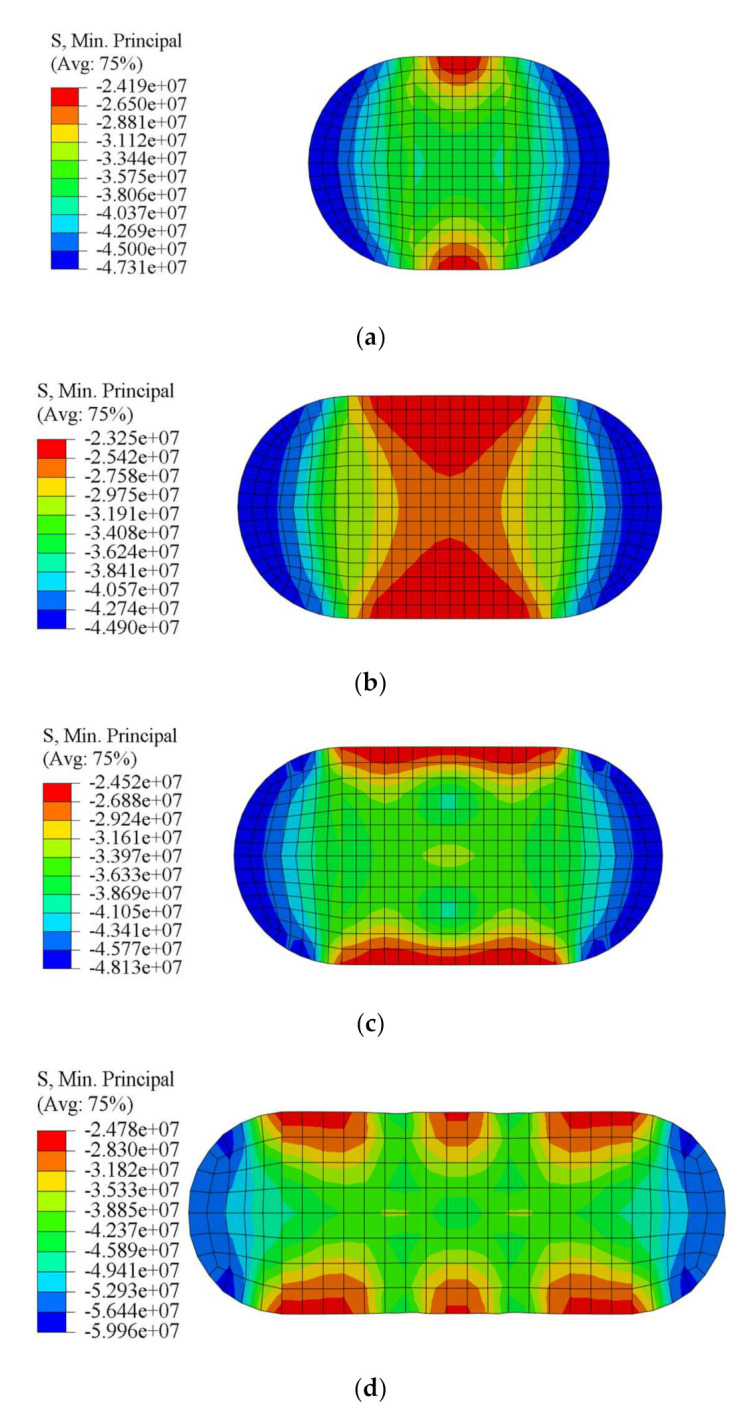
Stress diagram at mid cross-section of some specimens: (**a**) RRCFST-1; (**b**) RRCFST-3; (**c**) RRCFST-4; (**d**) RRCFST-13.

Since the concrete with no obvious yield point, the third principal stress, i.e., maximum compressive stress is adopted in this study to analyze the stress condition of the concrete. Some finite element results of concrete stress diagrams in the middle section are presented in Figure 11. The stress distribution of concrete is changed by tie bars; the low-stress areas with red color in the rectangular area are redistributed and reduced. It indicates that the confinement of steel tube on concrete is improved and the stress of the concrete is increased by adding tie bars.

### 3.3. Load-Deflection Relationship

The ultimate bearing load of the specimens are obtained and listed in Table 3. The results show that the addition of tie bars has a limited improvement on bearing capacity of columns with the small designed aspect ratios (*B*/*D* = 1.5, 2), which is an approximate 10% increase. The bearing capacity of RRCFST-8 with a large longitudinal spacing (*b*_s_) of the tie bars is lower than that of the specimens with a small *b*_s_ (RRCFST-5, RRCFST-7). For the columns with a large designed aspect ratio of 3, a single-limb tie bars can increase the bearing capacity by 15.4%, compared RRCFST-12 with RRCFST-11. However, bearing capacity cannot be further improved by adding more tie bars due to the concrete with low strength is more prone to shear and tensile damages. Increasing the diameter of the tie bars also result in the above phenomenon.

**Figure 12:**
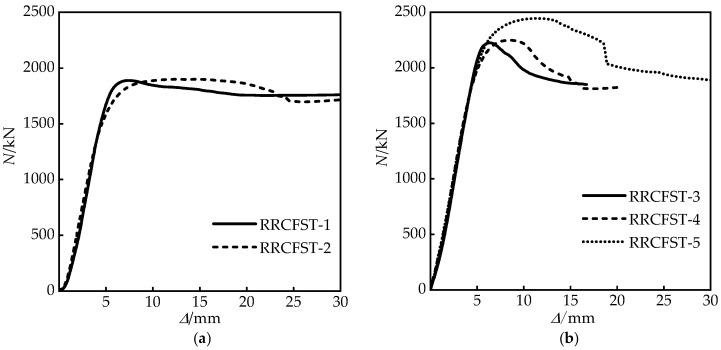

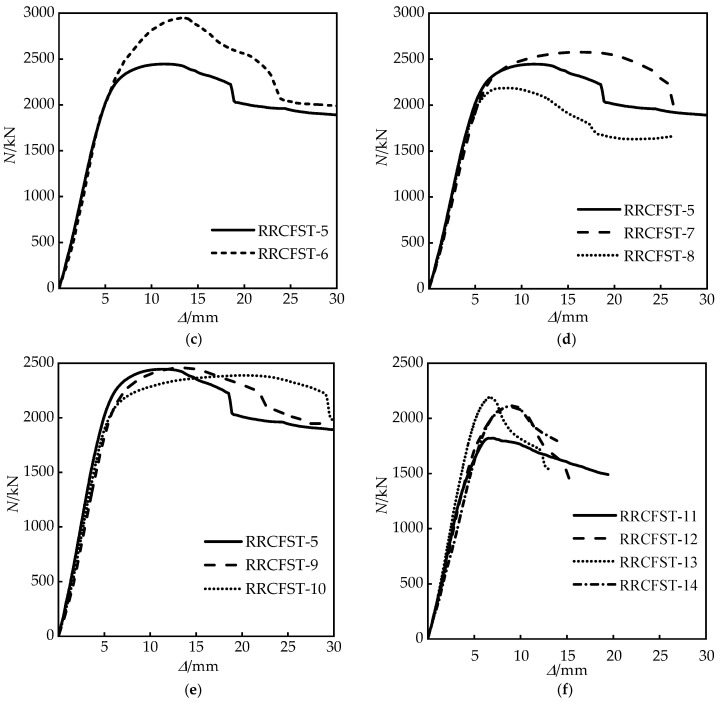
(**a**–**f**) Load–displacement curves of experiments.

Figure 12 presents load–displacement curves of each specimen. As can be seen in Figure 12a, for the columns with a designed aspect ratio of 1.5, the ultimate bearing capacity and the descent curve are slightly improved by the tie bars due to the limited confinement increasement, which is illustrated in Section 3.2. Figure 12b–e indicate that the tie bar has a large influence on the descent curve trend of the specimen with the designed aspect ratio of 2. The curves drop smoothly after the ultimate load, and the ductility can be improved when the number of tie bar limbs increases (Figure 12b), and a similar tendency is observed for the specimen with the designed aspect ratio of 3 (Figure 12f). According to the analysis in Section 3.2, the confined effect of steel tube (Figure 10b,c) and the section stress distribution (Figure 11b,c) of concrete are improved by tie bars. The stiffness of the specimen is promoted, which leads to the improvement of the development trend of the above curves. The descending stage of the curve (Figure 12d) is greatly affected by the longitudinal distance of tie bars (bs), the enveloping area under the curve is enlarged with an increased bs, which reflects that the longitudinal distance of tie bars has a great improvement on the ductility of RRCFST column. However, Figure 12e shows that the shape of the curve has a tiny relationship with the diameter of tie bars. By increasing the thickness of the steel tube (Figure 12c), the ultimate bearing capacity, ductility and energy absorption capacity of the test piece are significantly improved. In Figure 12f, curves of RRCFST-12 and RRCFST-14 share a similar trend. For RRCFST-14 with 3 limbs of tie bars, the middle tie bar has a more efficient contribution to bearing capacity than the two side tie bars due to the adequate utilization of tie bar deformation and strength, and curves 7, 8 in Figure 9i also illustrate this phenomenon.

### 3.4. Ductility

In this research, the ductility index *μ* is used to analyze the ductility of the RRCFST specimen. The displacement ductility index *μ*_Δ_ = *Δ*_u_/*Δ*_b_ is applied to characterize the inelastic deformation capacity of the specimen material after yielding [13]. *Δ*_u_ is the ultimate displacement of the specimen, usually taken as the displacement corresponding to the load drop to 0.85 *N*_u_. *Δ*_b_ = *Δ*_0.75_/0.75, and *Δ*_0.75_ is the displacement when the load attains 0.75 *N*_u_ before the peak load.

**Figure 13:**
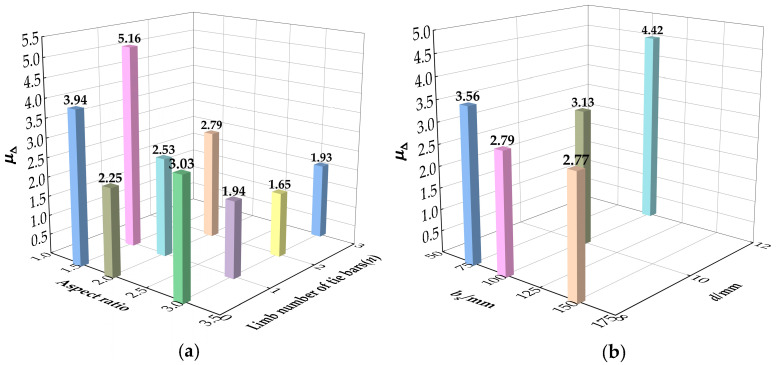
Displacement ductility: (**a**) With different aspect ratio and limb number of tie bars; (**b**) With different *b*_s_ and *d*.

Figure 13 presents the displacement ductility of the specimen under different parameters. As shown in Figure 13a, the inelastic deformation capacity of the columns with small aspect ratios increases greatly with an increase in the number of tie bar limbs. While for columns with high aspect ratios, the addition of tie bars has a negative impact on its ductility due to the larger loss of lateral stiffness, and the inelastic deformation capacity is weakened after the tie bars fail. Figure 13b shows the influence of different tie bar parameters on the ductility of the specimen. The longitudinal spacing and diameter of tie bars have a great promotion on their ductility. In particular, the diameter of tie bars changing from 8 mm to 12 mm greatly enhances displacement ductility by 58.4%. The deformation ability of the specimen is effectively improved with the reduced longitudinal spacing and increased diameter of the tie bars.

**Figure 14:**
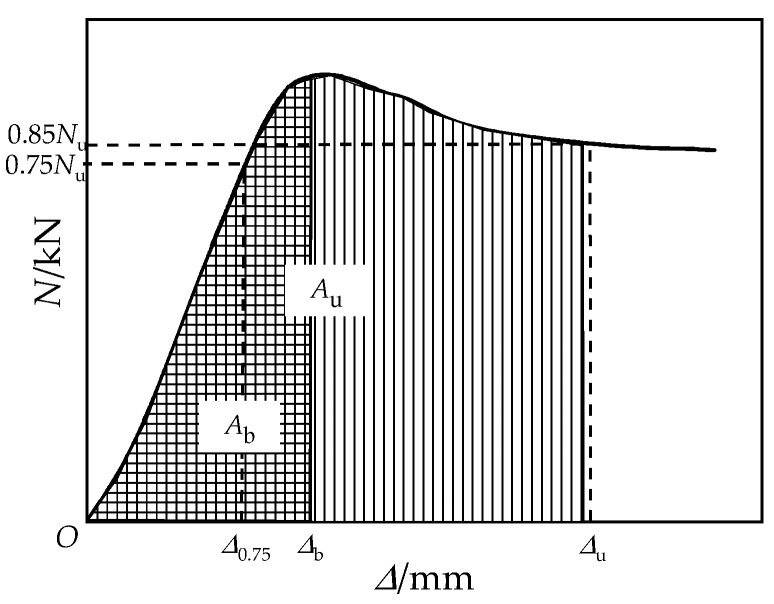
Typical load–displacement curve.

**Figure 15:**
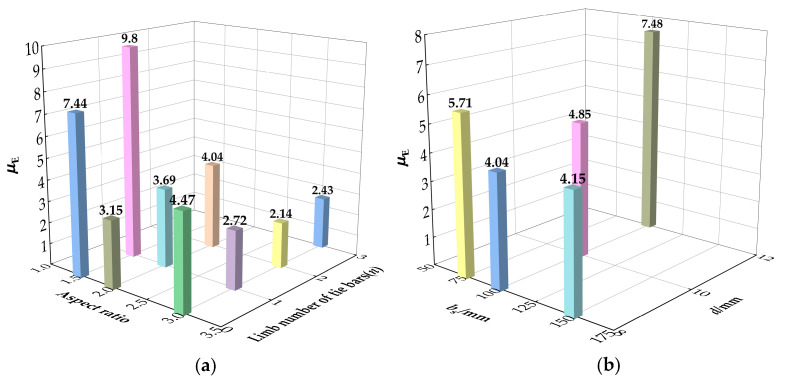
Energy ductility: (**a**) With different aspect ratio and limb number of tie bars; (**b**) With different *b*_s_ and *d*.

The ability of energy absorb for the RCFST columns can be described by energy ductility index *μ*_E_. The energy ductility index can be presented by *μ*_E_ = *A*_u_/*A*_b_, where *A*_u_ and *A*_b_ are the envelope areas under the load curve of the coordinate origin to the ultimate point and yield point, respectively [39]. Figure 14 demonstrates the regions of *A*_u_ and *A*_b_. The energy ductility of the columns shows a similar variation to the displacement ductility, as shown in Figure 15. Similarly, the diameter of tie bars from 8 mm to 12 mm had greatly enhanced energy ductility by 85.1%.

## 4. Analytical Model of Ultimate Bearing Load

Calculation methods of ultimate strength for rectangular and circular CFST are provided by current codes and specifications like AIJ [40], EC4 [41], AISC [42], GB50936 [43]. An analytical model is proposed to predict the ultimate strength of RRCFST with tie bars under axial compression, which is modified by reference [18]. The formula is expressed as:

(10) $$N_{u}=f_{cc}A_{c}+f_{y}A_{s}$$

where, *f*_y_ and *f*_cc_ are the longitudinal strengths of steel tube and confined concrete, respectively. *A*_s_ and *A*_c_ are the areas of steel tube and concrete, respectively. Based on reference [8], *f*_cc_ is defined by Equation (11), of which *f*_h_ is the confining stress.

(11) $$f_{cc}=f_{c}+3f_{h}$$

### 4.1. Confinement Model

**Figure 16:**
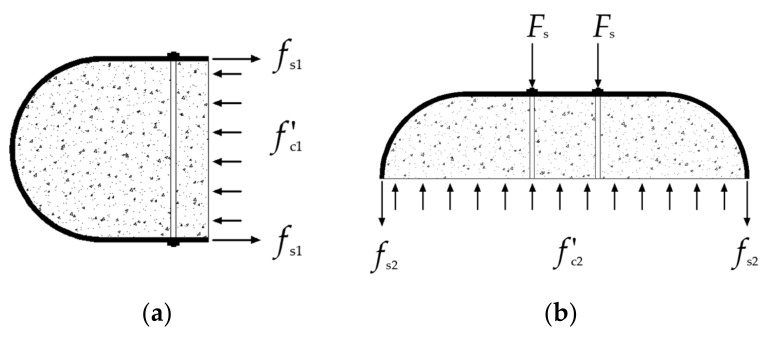
Interactions between the various components of the RRCFST with tie bars: (**a**) Round-ended part; (**b**) Rectangle part.

To describe the confined effect of steel tube and tie bars on the core concrete, the interactions between concrete and steel tube with tie bars are illustrated in Figure 16. This separate part with the depth along the longitude is equal to bs, which follow 3 hypotheses [8]:

(1) The size deflection of the structure can be neglected;

(2) Materials keep constant stresses beyond steel yielding stress and concrete ultimate compressive strain, and steel follows Von-Mises yield criterion;

(3) The core concrete is subjected to the homogenous compressive stress.

According to the experimental results in this paper, the tie bars arrive at around the ultimate strength before the column reaches ultimate strength. Therefore, *F*_s_ can be expressed by:

(12) $$F_{s}=f_{bu}A_{bs}$$

where *A*_bs_ is area of one tie bar.

The equilibrium between various forces can result from Figure 16 as follows:

(13) $$f^{\prime }{}_{c1}(D-2t)=2{tf}_{s1}$$

(14) $$f^{\prime }{}_{c2}(B-2t)b_{s}=2{tf}_{s2}b_{s}+{nF}_{s}$$

Of which:

(15) $$n=\frac{B-D}{a_{s}}-1$$

**Figure 17:**
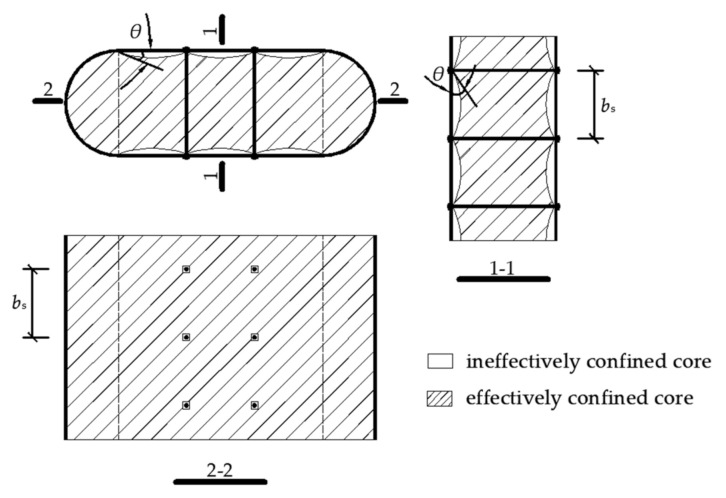
Effectively confined core of concrete for RRCFST with tie bars.

According to the stress diagram from finite element analysis in Section 3.2, the idealized partitions of effectively and ineffectively confined areas for the cross-section of RRCFST column with tie bars are presented in Figure 17, where *θ* is the initial tangent angle of the ineffectively confined area and given by Formula (16) [18].

(16) $$\theta =\frac{\pi }{180}(13+9.2a_{s}/100)$$

The confinement effectiveness coefficient of *k*_e1_ for round-ended part and *k*_e2_ for the rectangular part are considered for calculating the equivalent lateral confined stress *f*_c1_ and *f*_c2_. The formulas are defined by:

(17) $$f_{c1}=k_{e1}f^{\prime }{}_{c1}$$

(18) $$f_{c2}=k_{e2}f^{\prime }{}_{c2}$$

of which:

(19) $$k_{e1}=k_{e}k_{ea}$$

(20) $$k_{e2}=k_{e}\cdot 1$$

(21) $$k_{e}=1-\frac{(n+1)a_{s}^{2}tan\theta }{3A_{c}}$$

(22) $$k_{ea}={\left[1-\frac{b_{s}tan\theta }{2(D-2t)}\right]}^{2}$$

The local buckling strength (*f*_b_) of the steel plate is adopted to include the effects of local buckling. Based on the second hypothesis mentioned above, *f*_s1_ can be determined by Formulas (23)–(25) [18,35,36,43]. *R*_0_ is the depth-to-thickness ratio, the influence of local buckling can be ignored in the case of *R*_0_ < 0.85, and it proposes values of *f*_b_ = 0.89 *f*_y_ and *f*_s1_ = 0.19 *f*_y_ [18,43].

(23) $$f_{s1}=\frac{f_{b}-\sqrt{4f_{y}^{2}-3f_{b}^{2}}}{2}$$

Of which:

(24) $$f_{b}{}^{2}=(\frac{1.2}{R_{0}}-\frac{0.3}{R_{0}{}^{2}})f_{y}$$

(25) $$R_{0}=\frac{D}{t}\sqrt{\frac{12(1-v^{2})}{4\pi^{2}}}\sqrt{\frac{f_{y}}{E_{s}}}$$

Steel tube at round-ended part reaches yield stress at the ultimate status, and *f*_s2_ can be calculated as [22]:

(26) $$\frac{f_{s2}}{f_{y}}=\left\{\begin{array}{ll} 0 & 0\leq \;\xi <1/75\\ 0.15\xi -0.002 & 1/75\leq \xi <6.68\\ 1 & \xi >6.68 \end{array}\right.$$

The cross-section of the RRCFST can be simplified as a circle with an equivalent effectively confined concrete area and the steel ratio of RRCFST. The external and internal radiuses of the equivalent cross-section are *R* and *r*, respectively, and *f*_h_ take the form as:

(27) $$f_{h}=\frac{t(|f_{c1}|+|f_{c2}|)}{R+r}$$

Of which:

(28) $$\pi r^{2}=A_{c}-\frac{(n+1)a_{s}tan\theta }{3}$$

(29) $$\frac{\pi R^{2}-\pi r^{2}}{\pi r^{2}}=\frac{A_{s}}{A_{c}}$$

### 4.2. Verification

The calculated ultimate strengths for RRCFST columns with and without tie bars through Equation (10) are compared with the experimental results, as shown in Table 4. The average ratio of *N*_c_/*N*_u_ is 1.104, with a variation coefficient of 0.079. A good agreement is presented between calculated and experimental results of bearing capacity.

## 5. Conclusions

This paper experimentally and numerically investigates the effect of tie bars on axial compressive behavior of round-ended rectangular concrete-filled steel tube (RRCFST) stub columns. The failure mode, confined effect, load-deflection relationship, ductility and analytical model are researched and analyzed with different key design parameters. The main conclusion can be drawn as follow.

(1) The different constitutive models of concrete exerted at different regions are reliable. By considering the confined effect of tie bars, a modified constitutive model of concrete in a rectangular area is conducted and verified by experimental results.

(2) The RRCFST columns with small aspect ratios have good confinement of steel tube, which the material properties are sufficiently used. The lateral stiffness and buckling mode of RRCFST column with a relatively large aspect ratio can be effectively improved by tie bars. However, more tie bars (e.g., two or three limbs) utilization could not further improve the bearing capacity and failure mode. Reducing the longitudinal spacing of the tie bars can partially compensate for the local buckling, but the influence of the diameter of tie bar on buckling is not obvious.

(3) The effect of tie bars on the bearing capacity of RRCFST column with small aspect ratios (*B*/*D* = 1.5, 2) is not obvious, within approximately 10% increment. Concrete with low strength is more prone to shear and tensile damages when the limb number and diameter of tie bars are increased. The parameter matching optimization between concrete strength and tie bars is worth studying.

(4) The limb number, longitudinal span and diameter of tie bars have a large improvement on the displacement and energy ductility of RRCFST columns. Especially, the displacement and energy ductility increase 41.6% and 85.1%, respectively, with the diameter of tie bar changing from 8 mm to 12 mm. For the columns with high aspect ratios, the addition of tie bars has a negative impact on its ductility due to the larger loss of lateral stiffness, and the inelastic deformation capacity is weakened after the tie bars fail.

(5) An analytical model to calculate ultimate strength is proposed and validated by the results of experiments and FEM. This provides a feasible and reliable method for predicting the bearing capacity of RRCFST with tie bars.

## Figures and Tables

**Table 1:** Main parameter of the RRCFST columns.

Identifier	*D* × *B × L* (mm^3^)	*t* (mm)	*n × d* (mm)	*b*_s_ (mm)	*a*_s_ (mm)	*α*_s_
RRCFST-1	160 × 225 × 700	4	---	---	---	0.081
RRCFST-2	155 × 225 × 700	4	1 × 8	100	---	0.082
RRCFST-3	160 × 300 × 900	4	---	---	---	0.072
RRCFST-4	155 × 300 × 900	4	1 × 8	100	---	0.074
RRCFST-5	155 × 300 × 900	4	2 × 8	100	47	0.074
RRCFST-6	160 × 300 × 900	6	2 × 8	100	47	0.108
RRCFST-7	155 × 300 × 900	4	2 × 8	75	47	0.074
RRCFST-8	155 × 295 × 900	4	2 × 8	150	47	0.074
RRCFST-9	160 × 290 × 900	4	2 × 10	100	47	0.073
RRCFST-10	160 × 300 × 900	4	2 × 12	100	47	0.072
RRCFST-11	120 × 335 × 1000	4	---	---	---	0.086
RRCFST-12	120 × 330 × 1000	4	1 × 8	100	---	0.086
RRCFST-13	120 × 330 × 1000	4	2 × 8	100	70	0.086
RRCFST-14	120 × 330 × 1000	4	3 × 8	100	52.5	0.086

**Table 2:** Steel properties of the RRCFST.

Identifier	*t* or *d* (mm)	*f*_y_ (MPa)	*f*_u_ or *f*_bu_ (MPa)	*E*_s_ (GPa)	*γ*
Steel tube	4	254	406	240.0	0.274
Steel tube	6	290	440	230.0	0.275
Tie bar	8	---	448	177.6	0.290
Tie bar	10	---	426	169.1	0.290
Tie bar	12	---	455	170.8	0.290

**Table 3:** Comparison of EX and FE bearing capacity.

Identifier	*N*_u_ (kN)		FE/EX	*SI*	
	FE	EX		FE	EX
RRCFST-1	1825.27	1889	0.966	1.129	1.168
RRCFST-2	1927.16	1900	1.014	1.216	1.199
RRCFST-3	2318.04	2225	1.042	1.068	1.025
RRCFST-4	2359.01	2248	1.049	1.111	1.058
RRCFST-5	2415.4	2445	0.988	1.137	1.151
RRCFST-6	3234.52	2952	1.096	1.214	1.108
RRCFST-7	2467.04	2576	0.958	1.161	1.212
RRCFST-8	2340.01	2184	1.071	1.120	1.046
RRCFST-9	2473.23	2456	1.007	1.179	1.171
RRCFST-10	2581.34	2389	1.081	1.189	1.101
RRCFST-11	2069.66	1821	1.137	1.034	0.910
RRCFST-12	2075.03	2103	0.987	1.052	1.066
RRCFST-13	2166.79	2194	0.988	1.099	1.113
RRCFST-14	2203.02	2115	1.042	1.117	1.073

**Table 4:** Comparison of experimental and calculated bearing capacities.

Identifier	*N*_u_ (kN)	*N*_c_ (kN)	Ratio (*N*_c_/*N*_u_)
RRCFST-1	1889	1744.83	0.924
RRCFST-2	1900	1746.55	0.919
RRCFST-3	2225	2359.92	1.061
RRCFST-4	2248	2361.42	1.050
RRCFST-5	2445	2368.98	0.969
RRCFST-6	2952	2811.61	0.952
RRCFST-7	2576	2370.36	0.920
RRCFST-8	2184	2361.92	1.081
RRCFST-9	2456	2370.52	0.965
RRCFST-10	2389	2373.06	0.993
RRCFST-11	1821	2223.05	1.221
RRCFST-12	2103	2234.54	1.063
RRCFST-13	2194	2235.39	1.019
RRCFST-14	2115	2236.54	1.057

## Data Availability

The data presented in this study are available upon request from the corresponding author.
